# Carcass Yields and Physiochemical Meat Quality of Semi-extensive and Intensively Farmed Impala (*Aepyceros melampus*)

**DOI:** 10.3390/foods9040418

**Published:** 2020-04-03

**Authors:** Tersia Needham, Retha A. Engels, Daniel Bureš, Radim Kotrba, Berndt J. van Rensburg, Louwrens C. Hoffman

**Affiliations:** 1 Department of Animal Science and Food Processing, Faculty of Tropical AgriSciences, Czech University of Life Sciences Prague, Kamýcká 129, 16500 Prague-Suchdol, Czech Republic; needham@ftz.czu.cz (T.N.); kotrba@ftz.czu.cz (R.K.); 2 Department of Animal Sciences, University of Stellenbosch, Private Bag X1, Matieland, Stellenbosch 7602, South Africa; retha.engels@meadownatal.co.za; 3 Department of Cattle Breeding, Institute of Animal Science, Přátelství 815, 10400 Prague 10-Uhříněves, Czech Republic; bures.daniel@vuzv.cz; 4 Department of Food Quality, Faculty of Agrobiology, Food and Natural Sciences, Czech University of Life Sciences Prague, Kamýcká 129, 16500 Prague–Suchdol, Czech Republic; 5 Department of Ethology, Institute of Animal Science, Přátelství 815, 10400 Prague 10- Uhříněves, Czech Republic; 6 School of Biological Sciences, University of Queensland, Brisbane, QLD 4072, Australia; b.vanrensburg@uq.edu.au; 7 Center for Nutrition and Food Sciences, Queensland Alliance for Agriculture and Food Innovation (QAAFI), The University of Queensland, Health and Food Sciences Precinct, 39 Kessels Rd, Coopers Plains 4108, Australia

**Keywords:** impala, sex, production system, carcass yields, offal yields

## Abstract

The effects of sex and production systems on carcass yield, meat quality and proximate composition of sub-adult impala were evaluated by culling 35 impala from intensive (12 males) and semi-extensive (12 males and 11 females) production systems within the same game farm. While no sexual dimorphism was found for carcass weights, male impala had a higher dressing percentage than females, indicating a higher meat production potential. Few differences were observed for yields between the male impala from the different production systems, but physical meat quality parameters indicated possible stress for those kept intensively. Minor differences existed in physiochemical parameters between various impala muscles for the two sexes and production systems, providing little motivation for these factors to be considered when processing sub-adult impala carcasses. Impala meat from both sexes, all muscles and all production systems produced meat with shear force values below 43 N, and thus may be considered as tender. Furthermore, the proximate composition of all impala meat in this study ranged from 74.7 to 77.0 g/100g moisture, 20.7 to 23.5 g/100g protein, 1.2 to 2.2 g/100g fat and 1.1 to 1.3 g/100g ash content. These values compare favorably to other game species, indicating that impala meat may serve as a lean protein source.

## 1. Introduction 

Increasing knowledge of the economic and ecological sustainability of wildlife farming, combined with increasing financial incentives above that of traditional livestock farming, has resulted in the vast expansion of the South African wildlife (game) industry [1]. Subsequently, there has been a substantial shift in land-use allocation from farming traditional domestic livestock to indigenous game animals [2]. Indigenous antelope game species have evolved to be well-adapted to arid environments, with improved utilization of low-quality vegetation, lower susceptibility to overgrazing and better parasite and disease resistance than traditional domestic livestock [3]. Additionally, game animals are less susceptible to livestock theft as a result of the more stringent fencing requirements, larger camps and overall less domesticated nature than their domestic counterparts [4]. Game animals tend to generate higher net farm profit margins than livestock [2] and in comparison to live sales, trophy hunting and recreational hunting, game meat production generates the highest net revenue per biomass weight [5]. Due to the low intramuscular fat and high protein content of game meat [6,7,8], meat from indigenous South African game animals is also considered a healthy alternative protein source to red meat obtained from domestic livestock, and the culling of game through hunting is considered to be more humane than the present traditional livestock operations at abattoirs [9].

While game farming has typically involved extensive farming of animals with little to no intervention, the expansion of the game farming industry has been accompanied by the development of various production systems to optimize animal production according to the environment and financial resources available. Intensive production systems have been defined as small to moderate predator-controlled camps that require high human intervention through provision of the majority (or all) of the animals’ feed, water and healthcare requirements [1]. Semi-extensive game production systems are defined as environments large enough for the maintenance of self-sustaining game populations, regardless of fencing or lack thereof, but moderate human intervention is necessary in terms of feed supplementation, water and healthcare provision and/or parasite and predator control [3]. Semi-extensive game farming may encompass both intensive and extensive camps and may also include livestock farming. While extensive production systems are less labor-intensive, the practice of selective breeding has caused an increase in the utilization of intensive and semi-extensive production systems on game farms that aim to produce superior animals with higher sale values, such as rare color variants or animals with exceptional horn characteristics for trophy hunting. The consequent intensification of these production systems and the expansion in breeding of high-value color variants, such as black impala, has resulted in a surplus of so-called “split” animals (recessive gene carriers of color variant genes) and animals who are not selected for breeding, trophy hunting or live sale, which are typically culled for meat production. Currently, phenotypic color variant animals are maintained as high-value breeding stock or for hunting, and surplus animals which do not show the desirable phenotype are rather culled for meat production than sold live. At approximately 15 to 18 months of age, sub-adult male breeding replacement impala (regardless of color) are selected, as their horn size at this age is a good indication of their trophy potential, and those that do not meet the requirements for either trophy hunting or breeding at this age are culled for meat production. As the importance of the utilization of meat and offal generated from trophy hunting is receiving greater attention, to fully utilize each animal to its maximum potential, the muscle and offal yield of animals at this age are thus important factors to consider. 

Typically, antelope species produce higher lean meat yields than their domestic counterparts [10] as they deposit little (if any) subcutaneous fat. Game animals are currently sold per kilogram, as no grading system has been developed, and thus carcass yield is currently the most important economic performance parameter for game farming [3]. Antelope species also tend to show higher dressing percentages than domestic livestock, which is indicative of a lower offal and higher meat yield, but dressing percentages can vary significantly between game species due to differences in internal and external offal yield [7]. Of the antelope farmed in South Africa, impala meat is a popular choice for game meat export, with a contribution of approximately 20,858 kg exported in 2008 [11]. Impala have a wide geographical distribution throughout Africa (South Africa, Angola, Rwanda, Uganda and Kenya), rapid reproductive rate and relative abundance as a species that make them ideal for sustainable culling regimes [12,13,14]. Thus, impala is recorded as the most common herbivore species on South African game ranches, accounting for 24.1% of all animals counted [1]. Furthermore, the incorporation of impala improves the production potential of southern African farms when combined with beef cattle production [15]. Recently, the potential of impala for meat production has motivated research to expand toward factors influencing impala carcass yield [6,7,13,16], muscle characteristics [17] and meat quality [18,19]. However, none of the previous studies have compared different production systems within the same region, particularly ones using higher intensity of management (intensive/semi-extensive). 

Despite the suitability of impala for sustainable culling and the expansion of the market for game meat, the effect of production systems on the carcass yield and meat quality of this species has not yet been quantified. The aims of this study were, firstly, to compare the effect of sex on the carcass yields and physiochemical meat quality of impala from the same farm and production system, and secondly, to compare the effect of two different production systems from the same farm on the performance of male sub-adult impala. 

## 2. Materials and Methods 

### 2.1. Experimental Locations, Production Systems, Animals and Design

A total of 35 sub-adult (15–18 months) impala were culled (Research Ethics Committee: Animal Care and Use of Stellenbosch University Ethical Clearance Number: 10NP_HOF02) during autumn from one farm in Modimolle (Limpopo, South Africa). The farm in Modimolle is located within a summer rainfall area in the Central Sandy bushveld bioregion of the Savanna biome. Autumn is the period when the animals are at their peak body condition and lambs are old enough to be weaned. The rams were also old enough for evaluation of their horn size and shape for their trophy/breeding potential with inferior breeding stock being culled or hunted. The vegetation in this area varies with the landscape and includes *Acacia*, *Euclea* and *Ziziphus* species, with *Burkea africana* and *Terminalia sericea* as prominent deciduous woodland species and *Panicum maximum* as a dominant grass species [20]. Of the 35 impala, 24 were sub-adult males (±15–18 months old) culled from two production systems on the same farm, classified as intensive (*n* = 12 males) and semi-extensive (*n* = 12 males). Within the same semi-extensive production system, a further 11 females were culled to compare with the males harvested from the same semi-extensive production system. Males were aged according to horn size and shape, and as the females do not have horns, dentition was used to estimate their age. As impala have a very specific and short lambing season, the females were either 15–18 months or 27–30 months old.

The intensive system was a 0.25 ha paddock/boma system which required a high management input and contained only 12 male impala who were fed ad libitum with a total mixed ration within the boma for six months prior to slaughter, with no access to natural vegetation. Samples of the feed supplied in the intensive system were collected for chemical analysis (*n* = 3) and analyzed according to the Association of Official Analytical Chemists International (AOAC) official methods [21] for moisture (934.01), crude protein (992.15), ash (942.05) and fiber components (962.0). The feed had an as-is composition of 91.7% dry matter (8.3% moisture), 13.3% crude protein, 7.6% ash, 27.9% crude fiber, 47.7% neutral detergent fiber (NDF) and 30.5% acid detergent fiber (ADF). These nutritional values are within the recommended dietary requirements of less than 40% crude fiber and high protein content (8% in winter and 16% in summer) for this species [22]. 

The semi-extensive production system consisted of a 200 ha camp system and required moderate human intervention through supplementary feed (using the same feed as that supplied in the intensive system), although the primary feed intake of impala from this system was from grazing and browsing the natural vegetation in the Savanna biome. The semi-extensive system had a stocking density of approximately 250–300 animals in the 200 ha camp, with no apparent signs of overgrazing observed during culling. 

### 2.2. Culling, Carcass Processing and Yields

All impala were culled during the day (ethical clearance number 10NP_HOF02) using suppressor-equipped light caliber rifles (22 or 243) by head-shots. The impala were culled from a vehicle similar to that from which they had been fed and had become habituated to in order to minimize ante-mortem stress. The boma animals were culled from outside the boma through a specially designed viewing portal and so had no knowledge of the presence of the sharpshooter, although it was noted that these animals became more restless as the culling proceeded, most probably because they were in such a small enclosure. After shooting, the impala were exsanguinated in the field and immediately transported a short distance to the on-farm slaughter facility. At the slaughter facility, the undressed carcass weight (full body weight) of the exsanguinated impala carcasses were recorded. Thereafter, the impala were skinned, eviscerated and dressed [23]. The external offal (head, feet and skin) were removed and weighed first, followed by measurement of the horns. The internal offal was removed, and organ weights were recorded, with hearts and kidneys weighed after removal of the surrounding fat layers. Testes of male impala were weighed without the skin, and udders of females were weighed separately. The offal weights were expressed as a percentage of the full body weight to determine their relative yields. After dressing, the warm carcasses were weighed, and dressing percentages were calculated. After 24 h of cooling at ~4 °C, carcasses were weighed again to determine cold carcass weight, and thus moisture loss during chilling.

### 2.3. Physiochemical Meat Quality Evaluation

At 24 h post-mortem, six commercially important muscles were removed from both sides of the carcasses from the male and female impala for the comparison of sex: *longissimus thoracis et lumborum* (LTL), *biceps femoris* (BF), *semimembranosus* (SM), *semitendinosus* (ST), *infraspinatus* (IS) and *supraspinatus* (SS). For the production system comparison, only the LTL muscles were sampled for further analysis. All muscles were weighed individually and the ultimate pH at 24 hours post-mortem (pH_u_) of each impala muscle was measured within each muscle, using a calibrated Crison pH25 meter (Crison Instruments, Barcelona, Spain) fitted with a blade for easy insertion. Then, three ±2.0 cm-thick steaks were cut from the center of each sampled muscle for further physical analyses. Color measurements were taken with a calibrated Color-guide 45°/0° colorimeter (BYK-Gardner GmbH, Gerestried, Germany) at five random positions on the surface of the cut meat after a blooming period of 30 min. The measurements were in accordance with the International Commission on Illumination (CIE) Lab color system [24], which reported values according to lightness (CIE L*) from 0 (black) to 100 (white), red (–) to green (+) spectrum (CIE a*) and blue (–) to yellow (+) spectrum (CIE b*). The recorded CIE a* and CIE b* values were used for the calculation of the hue-angle (color definition) and chroma values (saturation/color intensity). Calculations were performed according to the following equations:

(1) $$\mathrm{Hue}-\mathrm{angle}\;({}^{\circ})\;=\;\tan-1\left(\frac{b^{*}}{a^{*}}\right)$$

(2) $$\mathrm{Chroma}\;(C*)\;=\;\sqrt{\left(a^{*}{}^{2}+b^{*}{}^{2}\right)}$$

Drip loss determination was performed by suspending one steak of each sample in inflated bags (clear 25-micron 100 × 150 mm polyethylene bags) for 24 h, at 4 °C [25]. The weight of each raw meat sample was expressed as a percentage of the initial weight to represent the drip loss. For the determination of cooking loss, the second steak of each fresh meat sample was weighed to obtain an initial weight and placed inside a labelled thin plastic bag (clear 25-micron 100 × 150 mm polyethylene bag). The samples were subsequently placed into a preheated water bath set to a constant temperature of 80 °C for a period of 60 min [26]. The cooked samples were removed from the water bath, excess water was drained, and the samples were cooled at 4 ± 1 °C for six hours. Cooking loss was determined by expressing the final weight of the cooked meat sample as a percentage of the initial weight of the fresh meat sample prior to cooking in the water bath [25]. Tenderness of impala meat was measured by determining the Warner-Bratzler shear force (WBSF) of the cooked meat samples using an Instron Universal Testing Machine (Instron UTM, Model 2519–107, Massachusetts, United States) fitted with a Warner-Bratzler blade (5 kN capacity). After the measurement of cooking loss, six cylindrical cores (with a diameter of 1.27 cm) were removed from the center of each sample, ensuring that visible collagen tissue was excluded from the sampled cores. Each core was sheared at a right angle to the longitudinal axis of the muscle fibers with a Warner-Bratzler blade (triangular blade: 58.96 × 58.96 mm sides; 60.50 mm base; 1.05 mm thick) at a speed of 3.33 mm/s. The blade was fitted to an electronic scale that measured the peak force (N) required to cut through the sample. The tenderness of each muscle was obtained by calculating the mean of the six measurements taken per sample, with lower shear force values associated with more tender meat [25]. 

In addition to the physical analyses, the same muscles were sampled for proximate analysis and vacuum packaged in labelled plastic bags, after which, the samples were frozen at −20 °C until chemical analysis could be performed. The samples selected for proximate analysis were removed from the −20 °C freezer and placed in a 4 ± 1 °C chiller overnight to thaw prior to the homogenization process. Proximate analyses were performed in duplicate for each sample. The moisture content (g/100 g meat) of each sample was determined after drying at 100 °C for 48 h [21], after which the moisture-free samples were placed inside a furnace at 500 °C for 6 h [21] to determine the ash content (g/100 g meat) of each sample. The intramuscular fat (IMF) content (g/100 g meat) was determined using a rapid solvent extraction (chloroform/methanol in a 1:2 ratio) method [27]. Following the extraction of fat from each meat sample, the remaining filtrate was collected, dried at 60 °C, and ground to a fine powder for determination of the crude protein content (g/100 g meat). One gram of each sample was enclosed in a Leco^TM^ tinfoil sheet and analyzed in a Leco Nitrogen/Protein Determinator (FP528, Leco Corporation). The results of the analysis were obtained in the form of nitrogen percentage (% N) for each sample and multiplied by a 6.25 conversion factor to obtain the crude protein content (g/100 g meat) of each sample.

### 2.4. Statistical Analysis 

Data were analyzed with SAS statistical software version 9.4 (Stat 14.1, 2015, SAS Institute Inc., Cary, North Carolina, USA), using the General Linear Models (GLM) procedure to perform a univariate analysis of variance (ANOVA). The data were analyzed separately for the “treatment” effects of sex (11 females and 12 males from the semi-extensive system) and production system (12 males from intensive system and 12 males from semi-extensive system), using the relevant animals, and thus the interactions between sex and production system were not evaluated. The model used to evaluate the sex or production system “treatments” was:

Y_ij_ = μ_i_ + α_j_ + ε_ij_ (3)

where Y_ij_ is the treatment effect (either sex or production) response, μ_i_ is the overall mean, α_j_ is the treatment effect and ε_ij_ is the unexplained error. For the carcass performance evaluation, both the sex and production system experimental designs were completely randomized and thus both sex and production system were considered as fixed effects in their respective analyses, and animal as the random effect. For the physiochemical parameters, the experimental design of the sex and muscle comparison was a completely random split plot design. Sex served as the main plot factor and muscle (LTL, BF, SM, ST, IS and SS) served as the subplot factor. As the production system comparison only evaluated the LTL muscle, a completely random experimental design was followed, with the male impala culled at random for each production system. Data obtained for the physiochemical meat quality attributes of impala from both trials were analyzed using the GLM procedure and univariate ANOVAs. The Shapiro–Wilk test was performed on the standardized residuals from the model to test for deviation from normality. Fisher’s least significant difference was calculated to compare sex or production system means. A level of 5% was considered significant for all tests.

## 3. Results

### 3.1. Carcass and Offal Yields

The effect of sex and production system on the carcass and offal yields of impala are presented in Table 1. No differences were observed between male and female impala for full body weight, dressed carcass weights (warm or cold) and thus, moisture lost during chilling (~1.9%), but males had significantly higher mean dressing percentages than females. Female impala had higher (*p* = 0.05) total offal yields than male impala. Differences were found between the yields of the spleen, GIT (gastrointestinal tract with full stomach and intestines) and total internal offal of male and female impala. The GIT had the highest proportional contribution to the total offal yield for both sexes and had a greater yield in females than in males (*p* = 0.05). Testes and udder weight made a small contribution to the body weight of the male (~0.5%) and female impala (~1%), respectively. A small percentage of the full body weight is unaccounted for in each study group, which may be explained by the loss of small amounts of fecal matter, urine and blood during the slaughter process. No differences were found between the sexes for the muscle weight yields (Table 2).

There were no differences between the production systems for full body weights, carcass weights, dressing percentages and offal yields. The semi-extensive system impala had higher yields for the lungs and trachea (*p* = 0.05) and lower kidney yields (*p* = 0.009) than intensive impala males. 

### 3.2. Physiochemical Meat Quality

**Figure 1:**
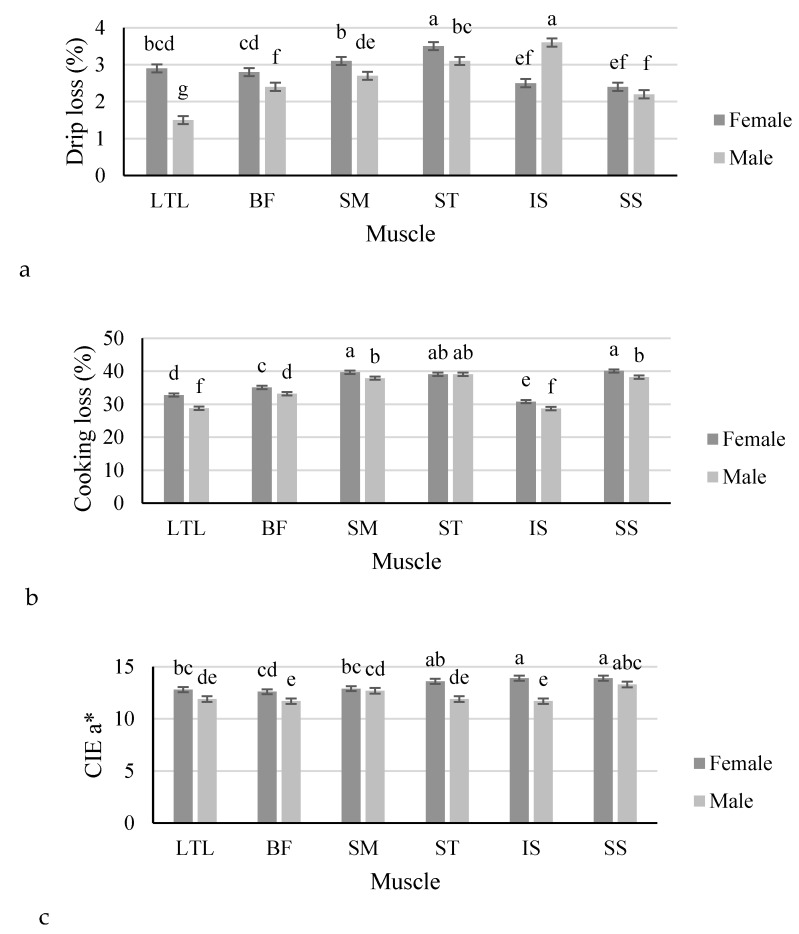
Interactions (LS Means ± standard error) between sex (12 males and 11 females) and muscle for (**a**) drip loss percentage, (**b**) cooking loss percentage and (**c**) CIE (International Commission on Illumination) a* color value. Abbreviations: LTL: *longissimus thoracis et lumborum*; BF: *biceps femoris*; SM: *semimembranosus*; ST: *semitendinosus*; IS: *infraspinatus*; SS: *supraspinatus*. Means (within a parameter) with different superscripts differ significantly from one another (*p* ≤ 0.05).

Interactions were observed between the effects of sex and muscle for drip loss percentage (*p* < 0.001), cooking loss percentage (*p* = 0.012) and the CIE a* values (*p* = 0.017) of impala meat (Figure 1). The drip loss percentage was higher in female impala for the LTL, BF, SM and ST muscles and lower in the IS muscles than in male impala, while drip loss percentage did not differ between sexes for the SS muscle (Figure 1a). The cooking loss percentage was also higher (*p* ≤ 0.05) in female impala for the LTL, BF, SM, IS and SS muscles than in male impala, while the sexes did not differ significantly for the cooking loss percentage of the ST muscle (Figure 1b). The a* values were lower (*p* ≤ 0.05) in male impala for the LTL, BF, ST, IS and SS muscles than in females, while no significant differences were recorded between sexes for the a* values of the SM muscle (Figure 1c). No further interactions were observed between sex and muscle for the remaining meat quality parameters of the impala. The main effects of sex and muscle are reported in Table 3. When comparing the influence of sex on the physical meat quality parameters of impala, the ultimate pH was higher (*p* = 0.021) in male impala than in females, but female impala produced meat with higher (*p* = 0.002) shear force values than male impala. The chroma values were also higher in meat from female impala than males (*p* = 0.05). Production system had a significant effect on the physical meat quality parameters of impala LTL muscles (Table 4). Impala from the intensive system had higher LTL pHu (*p* = 0.012), drip losses (*p* = 0.0006), cooking losses (*p* < 0.001) and shear force values (*p* < 0.001) than impala from the semi-extensive system. The CIE Lab, hue-angle and chroma values did not differ for the LTL meat color from the two production systems.

Interactions were found between sex and muscle for the moisture content (*p* = 0.002), protein content (*p* < 0.001), intramuscular fat (IMF) content (*p* < 0.001) and ash content (*p* = 0.004) of impala meat (Table 5). The moisture content (g/100 g) was higher in the BF, SM and SS muscles of male impala than in females, while female impala had a higher protein content in the LTL, SM and SS muscles than males. Female impala also had a higher IMF content than males for the BF muscle, whereas male impala had a higher IMF content for the LTL, SM, ST and SS muscles. The ash content did not differ significantly between sexes for the BF, SM, IS and SS muscles. However, the LTL muscle of male impala had a significantly higher ash content than females, while female impala had a higher ash content in the ST muscle than males. Production system had a significant influence on the proximate composition of the *longissimus thoracis et lumborum* (LTL) of sub-adult male impala (Table 4). The LTL protein (*p* = 0.0004) and IMF (*p* = 0.019) contents were higher in the intensive system impala. No differences were observed for both moisture and ash contents. 

## 4. Discussion 

### 4.1. Carcass and Offal Yields

Young impala (six months of age) showed no differences in carcass weights between sexes [28]; however, once sexual maturity has been reached at 13–16 months, it would be expected that male impala would have heavier live weights than females at the same age [29]. Sexual dimorphism for body weight has indeed been reported in mature impala [6,16,28,30,31]. However, there were no significant differences found between the mean full body or dressed carcass weights of male and female impala in the current study, despite their age (15–18 months for the males and 15–30 months for the females). Nonetheless, production of carcasses is typically focused within an optimum but narrow range of carcass weights, depending on the species. Thus, comparison the yields of male and female impala of similar carcass weights is beneficial.

The dressing percentage results obtained from the present study are similar to those previously recorded for sub-adult male impala (57.0%–59.2%) [16]. The mean pooled warm dressing percentage of male and female impala (57.4%) from the present study is higher than the dressing percentages of domestic livestock, which has been reported to range from 50.3% to 53.8% for cattle [32] and 41.5% to 44.2% for sheep [33]. In combination with the lack of visible subcutaneous fat in impala, these high dressing percentages are indicative of higher lean meat production potential in impala. 

The internal and external offal of impala is considered to be an edible by-product of meat production and may provide a wholesome and cost-efficient alternative protein source that may contribute to food security in Africa, with one impala yielding as much as three kilograms of edible offal [11]. In the present study, female impala had higher total offal yields than males, despite the males having higher total external offal yield, due to the significantly higher GIT yield of the females. Although gut-fill has an impact on the GIT yield, higher empty GIT yields of female impala compared to males has been reported [6,31]. Similarly, these studies also reported heavier head (mainly due to the horns being heavier in mature males) and feet yields of male impala [6,31]. 

When comparing impala from different production systems, it would be expected that impala produced in an intensive system should have higher live and carcass weights than those of animals raised in the semi-extensive system, at the same age. However, little difference was seen in the weight and yields of the impala harvested from the two different systems and the pooled means of sub-adult male impala were 39.7% total offal, which is comprised of 25.2% internal offal and 14.5% external offal. As the impala in the boma were provided with ad libitum supply of feed, this suggests that perhaps the intensively farmed impala were most likely under unspecified stress of captivity.

### 4.2. Physiochemical Meat Quality

Sex affected the ultimate pH of all sampled muscles in the present study, which is in accordance with previous findings, where the ultimate pH values of males (5.8 ± 0.13) were also significantly higher than that of female impala (5.7 ± 0.07) at 24 h post-mortem [34]. These differences in muscle pH between sexes may be caused by the higher vigilance behavior of male impala and consequently more dynamic reactions to any disturbance than that of female impala [35]. The pH_u_ of the different skeletal muscles in the present study ranged from 5.6 to 5.9, with differences between muscles most likely being the result of differences in function and activity levels [36]. Nonetheless, the pH_u_ of all impala muscles in the present study were within that range deemed normal in beef [37] and were also in accordance with previous findings recorded for impala [34]. 

The impala harvested from the intensive system within the present study had a higher LTL pH_u_, despite being culled within five minutes from the first shot in the 0.25ha boma. Thus, hunting stress may be excluded as a potential stressor, further indicating that the intensive management of the male impala within the boma was likely the contributing factor to stress and a higher ultimate pH in their meat. The impala were confined within the boma for six months, during which, some degree of habituation should have taken place; therefore, stocking rate is perhaps the contributing factor to the apparent stress of the male impala in the system and should be evaluated. 

The pH_u_ influences a number of other meat quality characteristics, largely pertaining to water-holding capacity, with a higher than normal pH_u_ often resulting in dark, firm and dry (DFD) meat [36,38]. Furthermore, when high amounts of moisture are lost within packaged meat, the liquid is negatively perceived by consumers and should therefore be minimized [39]. The drip loss percentages of male and female impala and intensive system impala in the present study were similar to the 2.5% to 2.9% range reported for night-culled impala in previous studies [6,41]. Despite the differences in drip losses for the LTL and IS muscles of male impala compared to females in the present study, there were minor differences in surface color and cooking losses between the muscles and sexes. Female red deer (*Cervus elaphus*) with low pH_u_ values were found to have meat that was redder and more saturated than meat from male deer, while male deer were observed to have higher pH_u_ values than females [41]. The higher redness and saturation in the meat of female impala in the present study may therefore also be the result of the lower pH_u_ recorded in female muscles. With the exception of the a* values and chroma values, the lack of differences between sexes for the surface color of impala meat is in accordance with previous research [34] and the less red color of the high-value LTL and BF muscles in this study may be the result of the lower oxymyoglobin content, color stability and decreased metmyoglobin-reducing activity associated with these muscles [42]. Nonetheless, the meat surface color values for the different sexes, muscles and production systems are in accordance to those considered normal for game meat [43].

Low meat cooking loss is associated with improved meat quality and greater juiciness in cooked meat due to higher amounts of moisture being retained in the meat [44]. The cooking loss percentages of the meat harvested from intensively produced impala was approximately 7% greater than that obtained from the semi-extensive impala, likely due to differences in pH, which is indicative of ante-mortem stress and had an influence on the subsequent shear force of their meat. However, the cooking loss percentages of impala meat from both sexes, all muscles and all production systems in this study are similar to the values obtained for impala meat by most previous research, with previous cooking loss percentages [40]. When comparing the shear force values between sexes, female impala produced meat with higher shear force values and thus less tender meat than male impala in the present study. This is in contrast to previous research findings, where no differences were recorded between male and female impala for meat tenderness [34]. The less tender meat of female impala compared to males in the present study may be explained by the age difference found between the sexes, where the female impala were estimated to be older than the male impala of the sex comparison. The skeletal muscle characteristics may explain the differences in tenderness between the various muscles of impala in the present study, where muscles from the hindquarters and back (SM and BF, followed by the ST and LTL) of impala had the highest shear force values (range of 25.5 to 31.7 N) and the IS muscle from the forequarter had the lowest shear force values (19.2 ± 1.10), while the SS muscle did not differ significantly from the LTL and ST muscles. This may be due to the fact that the muscles from the hindquarter and back are coarse-grained muscles that are frequently used for exercise, while the more tender IS muscle has a more supportive function. When comparing the tenderness of the LTL of both male and female impala in the present study to the shear force values obtained in previous research, impala meat was found to be similar to that mountain reedbuck (*Redunca fulvorufula*) [45] and less tender than springbok (*Antidorcas marsupialis*) [46]. Impala meat therefore compares favorably to meat from other game and domestic species.

The intensive system impala produced the least tender meat of the production systems, which may be attributed to the consequence of increased muscle pH_u_. When compared to previous research, impala from the intensive boma system had substantially higher shear force values than night-culled male impala from Maneze Wildlife Conservancy in Zimbabwe [34]. Despite variation in shear force values between sexes, muscles and production systems, the meat of impala from both sexes and all muscles and production systems in this study are classified as being tender overall due to all mean shear force values (range of 22.4 to 39.3 N) falling below the stipulated maximum of 42.9 N [47].

The proximate composition of the various impala muscles for the two sexes and two production systems in the present study showed minor differences and were comparable to results obtained from previous studies on impala. Typically, lean skeletal muscles generally have a biochemical composition of approximately 75 g/100 g meat moisture, 20 g/100 g meat protein, 1–10 g/100 g meat IMF and 1 g/100 g meat carbohydrates, vitamins and minerals, with the latter usually analyzed as ash [48,49]. The moisture content of all impala meat in the present study ranged from 74.7 to 77.0 g/100 g. Previous studies on the moisture content of impala meat were limited to the LTL muscle, thus making it difficult to compare the proximate values obtained for all muscle types. Even so, the moisture content of only the LTL impala meat in the present study was comparable to the values previously obtained for the LTL muscles of impala, which ranged from 70.2 to 75.7 g/100 g [6,7,8]. When compared with the LTL muscles of other game species, the moisture content of impala meat in the present study was higher than that recorded for springbok (65.3–65.8 g/100 g) [50] and similar to that of blesbok (*Damaliscus pygargus phillipsi*) (73.9–76.1 g/100 g) [51], but slightly lower than common eland (*Taurotragus oryx*) [24]. The protein content of all impala in the present study ranged from 21.4to 23.4 g/100 g, which is comparable to the values previously recorded for impala LTL muscles [6] and similar to blesbok (19.0–23.1 g/100 g) [51] but lower than springbok (31.1 ± 0.45 g/100 g) [50]. 

Game meat is characterized as a low-kilojoule, low-fat product with an intramuscular fat (IMF) content that generally falls below three percent [52]. The IMF content of meat is an important characteristic of the meat quality due to its relation to tenderness and flavor of meat, particularly during cooking or heat treatment [53,54,55]. Production system was found to have a very slight effect on the IMF content of meat from sub-adult male impala in the present study, likely due to differences in dietary energy, as the intensively farmed impala received a commercial feed ad libitum. The IMF content of meat from impala of both sexes, all muscles and all production systems in the present study ranged from 1.3 to 2.0 g/100 g, and is similar to eland (1.45–1.48 g/100 g) [24] but lower than that of blesbok (2.3–3.4 g/100 g) [51]. The low IMF content of impala meat indicates the leanness of meat from this game species, which makes it an appealing option for health-conscious consumers.

## 5. Conclusions

Male impala showed greater meat production potential than females due to their higher dressing percentages; however, the dressing percentages for all impala in the study were superior to those of domestic livestock. Contrary to expectation, the use of an intensive finishing system presented no substantial advantage in terms of carcass yields over that of the semi-extensive system when impala are harvested at 15–18 months of age. Thus, the boma management strategy of impala should be further investigated together with the monitoring of stress responses. While the physical meat quality parameters of impala meat were influenced by sex, muscle and production system, these differences were mostly marginal and should be evaluated by a trained sensory panel to motivate whether it is necessary to discriminate between them for impala carcass processing. Similarly, the proximate composition differences were minor and numerically too small to provide a substantial nutritional advantage for meat produced from different sexes, muscles and production systems. Overall, this study found all impala meat to be high in protein and low in intramuscular fat content and compared favorably to traditional livestock species and other species of game. In conclusion, impala meat can be classified as tender with appealing qualities for health-conscious consumers that can serve as an alternative to traditional red meat.

## Figures and Tables

**Table 1:** Least Square (Means ± standard error) of impala carcass and offal yields (expressed as percentage of undressed carcass weight) as influenced by sex and production system.

Parameters	Sex			Production System		
	Female (*n* = 11)	Male (*n* = 12)	*p*	Intensive (*n* = 12)	Semi-Extensive (*n* = 12)	*p*
Undressed carcass (kg)	37.8 ± 1.30	36.4 ± 1.30	0.451	37.9 ± 0.92	36.4 ± 0.96	0.287
Warm dressed carcass (kg)	21.0 ± 0.82	21.6 ± 0.82	0.639	21.9 ± 0.65	21.3 ± 0.68	0.500
Warm dressing percentage	55.6 ± 0.76	59.1 ± 0.76	0.004	57.9 ± 0.51	58.4 ± 0.54	0.505
Cold dressed carcass (kg)	20.5 ± 0.82	20.9 ± 0.82	0.727	20.9 ± 0.65	20.7 ± 0.67	0.789
Total offal (%)	41.6 ± 0.84	39.2 ± 0.84	0.05	39.4 ± 0.42	40.4 ± 0.43	0.130
Total external offal (%)	12.5 ± 0.28	14.8 ± 0.28	<0.001	14.6 ± 0.24	14.7 ± 0.25	0.638
Total internal offal (%)	29.1 ± 0.88	24.3 ± 0.88	<0.001	24.9 ± 0.40	25.6 ± 0.39	0.156
Head (%)	5.1 ± 0.12	6.5 ± 0.12	<0.001	6.64 ± 0.07	6.56 ± 0.08	0.506
Feet (%)	2.8 ± 0.08	3.3 ± 0.08	<0.001	3.3 ± 0.12	3.2 ± 0.13	0.817
Skin (%)	4.6 ± 0.20	5.0 ± 0.20	0.192	4.6 ± 0.18	4.9 ± 0.19	0.294
Heart (%)	0.7 ± 0.01	0.7 ± 0.01	0.139	0.7 ± 0.02	0.7 ± 0.02	0.111
Lungs & trachea (%)	1.9 ± 0.15	2.1 ± 0.15	0.312	1.8 ± 0.11	2.1 ± 0.12	0.05
Liver (%)	1.6 ± 0.05	1.6 ± 0.05	0.451	1.4 ± 0.05	1.6 ± 0.05	0.059
Kidneys (%)	0.3 ± 0.01	0.3 ± 0.01	0.072	0.31 ± 0.01	0.27± 0.01	0.009
Spleen (%)	0.4 ± 0.04	0.5 ± 0.04	0.018	0.47 ± 0.02	0.53 ± 0.02	0.106
GIT (%)	24.3 ± 0.82	18.9 ± 0.82	<0.001	19.9 ± 0.47	20.5 ± 0.49	0.416

GIT = gastro-intestinal tract (includes full stomach and intestines).

**Table 2:** Least Square (Means ± standard error) of commercially important impala muscle weight (g) as influenced by sex.

Muscle	Sex		*p*
	Female (*n* = 11)	Male (*n* = 12)	
LTL	855.4 ± 34.41	850.1 ± 34.41	0.916
BF	610.8 ± 27.38	602.2 ± 27.38	0.826
SM	638.9 ± 30.20	644.0 ± 31.68	0.910
ST	182.1 ± 7.28	183.0 ± 7.28	0.937
IS	164.6 ± 7.73	178.4 ± 7.73	0.222
SS	139.8 ± 6.78	154.0 ± 6.78	0.155

LTL: *longissimus thoracis et lumborum*; BF: *biceps femoris*; SM: *semimembranosus*; ST: *semitendinosus*; IS: *infraspinatus*; SS: *supraspinatus*.

**Table 3:** The effect of sex and muscle type on the physical meat quality parameters of impala meat (Least Square Means ± standard error).

Parameter	Sex			Muscle						
	Female (*n* = 11)	Male (*n* = 12)	*p*	LTL	BF	SM	ST	IS	SS	*p*
pH_u_	5.6 ± 0.05	5.8 ± 0.05	0.021	5.6 ± 0.03 ^c^	5.7 ± 0.03 ^c^	5.6 ± 0.03 ^c^	5.7 ± 0.03 ^c^	5.8 ± 0.03 ^b^	5.9 ± 0.03 ^a^	<0.001
Drip loss (%)	2.9 ± 0.08	2.6 ± 0.08	0.013	2.2 ± 0.08 ^d^	2.6 ± 0.08 ^c^	2.9 ± 0.08 ^b^	3.3 ± 0.09 ^a^	3.0 ± 0.08 ^b^	2.3 ± 0.08 ^d^	<0.001
Cooking loss (%)	36.3 ± 0.57	34.3 ± 0.59	0.028	30.8 ± 0.36 ^c^	34.1 ± 0.35 ^b^	38.8 ± 0.35 ^a^	39.1 ± 0.36 ^a^	29.8 ± 0.37 ^d^	39.2 ± 0.35 ^a^	<0.001
Shear force (N)	28.8 ± 1.08	23.2 ± 1.07	0.002	25.5 ± 1.10 ^b^	30.1 ± 1.10 ^a^	31.7 ± 1.13 ^a^	25.7 ± 1.10 ^b^	19.2 ± 1.10 ^c^	23.6 ± 1.10 ^b^	<0.001
Color:										
L*	33.5 ± 0.77	34.8 ± 0.77	0.247	31.4 ± 0.41 ^d^	34.1 ± 0.41 ^b^	32.8 ± 0.41 ^c^	36.8 ± 0.41 ^a^	36.1 ± 0.41 ^a^	33.9 ± 0.41 ^bc^	<0.001
a*	13.3 ± 0.27	12.2 ± 0.27	0.009	12.3 ± 0.21 ^bc^	12.1 ± 0.20 ^c^	12.8 ± 0.20 ^b^	12.7 ± 0.20 ^b^	12.8 ± 0.20 ^b^	13.6 ± 0.20 ^a^	<0.001
b*	8.4 ± 0.47	7.7 ± 0.47	0.280	7.3 ± 0.28 ^d^	7.6 ± 0.27 ^d^	8.1 ± 0.27 ^bc^	8.8 ± 0.27 ^a^	8.6 ± 0.27 ^ab^	8.1 ± 0.27 ^abc^	0.001
Chroma	15.8 ± 0.45	14.5 ± 0.45	0.05	14.5 ± 0.29 ^bc^	14.4 ± 0.28 ^c^	15.2 ± 0.28 ^ab^	15.6 ± 0.28 ^a^	15.5 ± 0.28 ^a^	15.9 ± 0.28 ^a^	0.001
Hue-angle	32.1 ± 1.09	32.0 ± 1.10	0.965	29.6 ± 0.77 ^b^	31.6 ± 0.75 ^b^	31.7 ± 0.75 ^b^	34.6 ± 0.75 ^a^	33.8 ± 0.75 ^a^	30.9 ± 0.75 ^b^	<0.001

Abbreviations: LTL: *longissimus thoracis et lumborum*; BF: *biceps femoris*; SM: *semimembranosus*; ST: *semitendinosus*; IS: *infraspinatus*; SS: *supraspinatus*; pH_u_: *ultimate pH*. L*: lightness from 0 (black) to 100 (white), a*: lightness from red (–) to green (+) spectrum, b*: lightness from blue (–) to yellow (+) spectrum.

**Table 4:** Least Square (Means ± standard error) of the *Longissimus thoracis et lumborum* (LTL) muscle’s physical meat quality characteristics of impala from two different production systems used on the same farm within the Limpopo region of South Africa.

Parameter	Production System		*p*
	Intensive (*n* = 12)	Semi-Extensive (*n* = 12)	
pH_u_	5.8 ± 0.04	5.6 ± 0.05	0.012
Drip loss (%)	2.2 ± 0.13	1.5 ± 0.14	0.006
Cooking loss (%)	36.8 ± 0.53	29.5 ± 0.57	<0.001
Shear force (N)	39.3 ± 1.78	22.4 ± 1.85	<0.001
Color			
L*	30.9 ± 0.77	32.2 ± 0.81	0.272
a*	11.4 ± 0.37	12.2 ± 0.38	0.145
b*	6.0 ± 0.57	7.1 ± 0.59	0.186
Chroma	13.1 ± 0.52	14.2 ± 0.54	0.121
Hue-angle	27.8 ± 1.73	29.4 ± 1.81	0.542
Proximate composition (g/100 g)			
Moisture	75.3 ± 0.13	75.7 ± 0.13	0.191
Protein	22.7 ± 0.12	22.0 ± 0.12	0.0004
Fat	2.0 ± 0.06	1.8 ± 0.06	0.019
Ash	1.21 ± 0.01	1.19 ± 0.01	0.117

Abbreviations: pH_u_: ultimate pH measured at 24 h post-mortem.

**Table 5:** Least Square (Means ± standard error) of the proximate composition (g/100 g) of six different muscles from impala (*n* = 22) as influenced by sex and muscle.

Parameter (g/100 g)	Muscle	Sex		*p*
		Female (*n* = 11)	Male (*n* = 12)	
Moisture	LTL	76.0 ± 0.15 ^cd^	76.0 ± 0.13 ^c^	0.002
	BF	75.2 ± 0.13 ^fg^	75.6 ± 0.13 ^de^	
	SM	74.8 ± 0.13 ^g^	75.3 ± 0.13 ^ef^	
	ST	76.1 ± 0.13 ^c^	76.0 ± 0.13 ^c^	
	IS	76.3 ± 0.13 ^bc^	76.5 ± 0.13 ^b^	
	SS	76.1^c^ ± 0.13	77.0 ± 0.13 ^a^	
Protein	LTL	22.6 ± 0.19 ^bc^	21.6 ± 0.18 ^ef^	<0.001
	BF	23.1 ± 0.18 ^ab^	22.7 ± 0.18 ^bc^	
	SM	23.5 ± 0.18 ^a^	22.3 ± 0.18 ^cd^	
	ST	22.6 ± 0.18 ^cd^	22.8 ± 0.18 ^bc^	
	IS	21.6 ± 0.18 ^ef^	21.4 ± 0.18 ^f^	
	SS	21.5 ± 0.12 ^c^	20.7 ± 0.19 ^g^	
Intramuscular fat	LTL	1.2 ± 0.08 ^f^	1.9 ± 0.08 ^b^	<0.001
	BF	1.6 ± 0.08 ^c^	1.3 ± 0.08 ^def^	
	SM	1.4 ± 0.08 ^cd^	2.2 ± 0.08 ^a^	
	ST	1.1 ± 0.08 ^f^	1.4 ± 0.08 ^cde^	
	IS	1.9 ± 0.08 ^b^	2.1 ± 0.09 ^ab^	
	SS	1.2 ± 0.08 ^ef^	1.8 ± 0.09 ^b^	
Ash	LTL	1.2 ± 0.02 ^b^	1.4 ± 0.03 ^a^	0.004
	BF	1.2 ± 0.02 ^b^	1.2 ± 0.02 ^bc^	
	SM	1.2 ± 0.02 ^bcd^	1.2 ± 0.02 ^bcd^	
	ST	1.2 ± 0.02 ^b^	1.2 ± 0.02 ^cde^	
	IS	1.1 ± 0.02 ^e^	1.1 ± 0.02 ^de^	
	SS	1.1 ± 0.02 ^cde^	1.1 ± 0.02 ^cde^	

LTL: *longissimus thoracis et lumborum*; BF: *biceps femoris*; SM: *semimembranosus*; ST: *semitendinosus*; IS: *infraspinatus*; SS: *supraspinatus*. ^a–g^ Means with different superscripts within a parameter differ significantly from each other (*p* ≤ 0.05).
